# Whole genome functional analysis identifies novel components required for mitotic spindle integrity in human cells

**DOI:** 10.1186/gb-2008-9-2-r44

**Published:** 2008-02-26

**Authors:** Daniel R Rines, Maria Ana Gomez-Ferreria, Yingyao Zhou, Paul DeJesus, Seanna Grob, Serge Batalov, Marc Labow, Dieter Huesken, Craig Mickanin, Jonathan Hall, Mischa Reinhardt, Francois Natt, Joerg Lange, David J Sharp, Sumit K Chanda, Jeremy S Caldwell

**Affiliations:** 1 Genomics Institute of Novartis Research Foundation, John Jay Hopkins Drive, San Diego, California 92121, USA; 2 Department of Physiology and Biophysics, Albert Einstein College of Medicine, 1300 Marris Park Avenue, Bronx, New York 10461, USA; 3 Novartis Institute for Biomedical Research Inc., Mass Avenue, Cambridge, Massachusetts 02139, USA; 4 Novartis Pharma AG, Postfach, CH-4002 Basel, Switzerland; 5 Infectious & Inflammatory Disease Center, Burnham Institute for Medical Research, North Torrey Pines Road, La Jolla, California 92037, USA

## Abstract

**Background:**

The mitotic spindle is a complex mechanical apparatus required for accurate segregation of sister chromosomes during mitosis. We designed a genetic screen using automated microscopy to discover factors essential for mitotic progression. Using a RNA interference library of 49,164 double-stranded RNAs targeting 23,835 human genes, we performed a loss of function screen to look for small interfering RNAs that arrest cells in metaphase.

**Results:**

Here we report the identification of genes that, when suppressed, result in structural defects in the mitotic spindle leading to bent, twisted, monopolar, or multipolar spindles, and cause cell cycle arrest. We further describe a novel analysis methodology for large-scale RNA interference datasets that relies on supervised clustering of these genes based on Gene Ontology, protein families, tissue expression, and protein-protein interactions.

**Conclusion:**

This approach was utilized to classify functionally the identified genes in discrete mitotic processes. We confirmed the identity for a subset of these genes and examined more closely their mechanical role in spindle architecture.

## Abstract 2

A loss-of-function screen for siRNAs that arrest human cells in metaphase reveals genes involved in mitotic spindle integrity.

## Background

Dynamic changes in spindle structure and function are essential for maintaining genomic integrity during cell division [1-3]. The mitotic spindle goes through several structural rearrangements, initiating with centrosome separation in prophase and concluding with midbody formation during telophase [4,5]. Failures in spindle functions have been linked with both aneuploidy and tumorigenesis [6-8]. Furthermore, a common phenotype of many cancer cell types is genomic instability, which is generally correlated with incorrectly assembled spindles [8-10]. Studies of spindle assembly have demonstrated that a variety of proteins play a role in these rearrangements, including microtubule-associated proteins, motor proteins, DNA-binding proteins, kinases, phosphatases, and even nonproteinaceous components [11-15]. These studies, however, have yet to reveal an exhaustive list of all of the members involved. Here, we describe the execution and analysis of an image-based RNA interference (RNAi) screen to functionally elucidate genes required for mitotic progression in a mammalian cell type. This study provides a novel methodology with which to integrate genome-wide RNAi screens with other large-scale functional datasets, including gene expression, Gene Ontology (GO), and protein-protein interaction. Importantly, this approach has resulted in the identification of more than 200 genes that putatively regulate the metaphase to anaphase transition.

## Results and discussion

**Figure 1:**
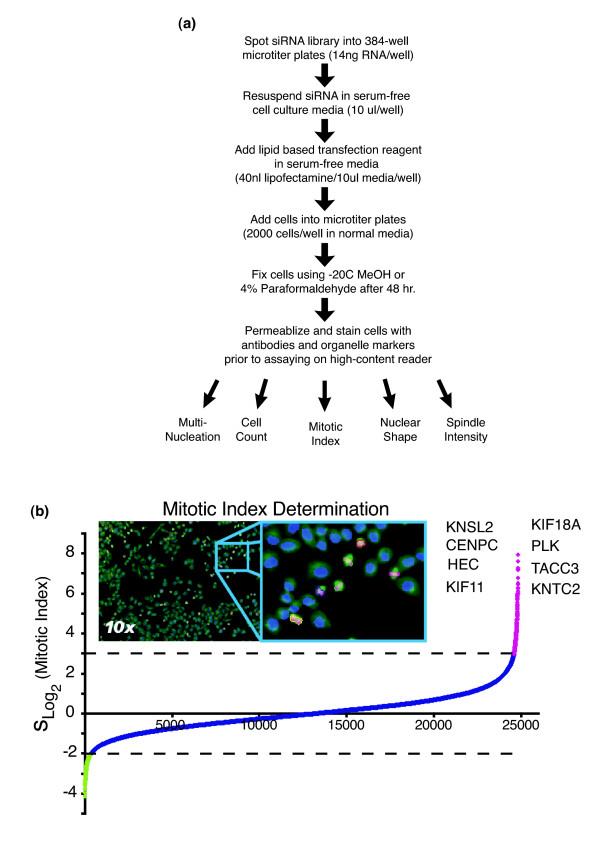
Genome-wide library screen analyzed for mitotic spindle genes. **(a) **Outline for transfecting cells in 384-well microtiter plate format. Small interfering RNAs (siRNAs) are arrayed into 384-well microtiter plates (two siRNAs/well) and mixed with a lipid-based transfection reagent. Cell transfections are performed in a reverse or (retro)transfection manner, in which the cell culture is added to the preformed siRNA/lipid complexes and incubated at 37°C for 48 hours before -20°C methanol (MeOH) fixation. Indirect immunolocalization is used to fluorescently label cells in metaphase based on phospho-histone H3 (pHis) activity. HeLa cells were transfected in 384-well microtiter plates with 49,164 synthetic double-stranded RNAs (dsRNAs). Cells were also fluorescently labeled for α-tubulin (green), pHis (red), and DNA (blue) before being imaged on an automated microscope. **(b) **Plate normalized mitotic index values for the knock-down of 23,835 genes in duplicate are plotted on a Log2 scale. Calculated values are sorted in rank order (lowest to highest) and represented by the curve. Target genes with mitotic index values -2σ below the mean are shown in green. Genes with values 3σ above the mean are shown in magenta. Genes with values between the thresholds are shown in blue. Upper and lower dashed lines indicate scoring thresholds. A partial list of previously characterized genes having an essential role in chromosome segregation is given.

Since mechanical defects in the spindle inhibit mitotic cell cycle progression, we set out to identify novel factors that affect spindle function by screening an small interfering RNA (siRNA) library targeting approximately 23,835 unique human genes [16]. In the first part of this screen, HeLa cells were transfected with pools of two double-stranded RNAs for each gene, based on methods described previously [17] (Figure 1a). To identify cells in mitosis, cells were fluorescently labeled using an antibody directed against the Ser10 phosphorylated form of histone H3 (pHis) 48 hours after the siRNA transfections. In addition, cells were fluorescently labeled for α-tubulin and DNA before being imaged on an automated microscope. We initially acquired 308,736 images and performed quantitative image analysis to establish a list of candidate genes by identifying cell populations with high levels of pHis staining (Figure 1b). Mitotic index values between 10% and 40% were observed when silencing many known spindle components; this is in contrast to normal cycling cultures, which typically demonstrate 5% mitotic cells. After normalizing for plate based variations, we observed that 226 genes were 3σs above the mean mitotic index, our cut-off threshold. (See Additional data file 1 for the complete list of gene annotations.) These data suggest that more than 1% of all human genes are specifically required for faithful mitotic spindle functions.

We were also able to assess additional morphologic parameters for each of the siRNA-treated cell populations. Nuclear fragmentation, cell shape, cellular proliferation, multinucleation, and fluorescent spindle intensity were some of the parameters recorded in our machine vision approach. (See Additional data file 2 for examples of other parameters measured during the image analysis steps.) We reasoned that genes with similar effects on morphologic parameters may be involved in common biologic processes. In order to cluster these genes functionally, we employed an ontology-based pattern identification (OPI) algorithm [18]. This algorithm was applied using two databases: the GO database, which is organized around biologic processes, cellular components, and molecular functions; and the InterPro database, which considers protein families, domains, and functional sites. OPI analysis of siRNAs sharing both unusually similar morphologic patterns and statistically significant enrichment in certain functional areas are identified by clustering.

From the complete list of 23,835 genes, we first identified representative morphology patterns based on known members of each functional group. We automatically determined a statistically optimized similarity threshold that associated novel genes from our screen with genes of known function. Among the 7,856 GO/InterPro groups examined, 445 survived iterative simulations (*P *≤ 0.05). Without any subjective cut-off values, the morphologic profiles of these 445 clusters were organized hierarchically and the relevant biologic information unveiled. We further carried out similar OPI analyses to highlight clusters that have unusually high numbers of interactions in either protein-protein interaction (PPI) networks based on a yeast two hybrid database (Prolexys), or rare co-expression profiles across 79 key human tissue samples [19]. (See additional data file 3 for the OPI cluster's data table supporting these connections.) These meta-analyses have created additional lines of evidence for the cellular role of our novel candidate genes.

**Figure 2:**
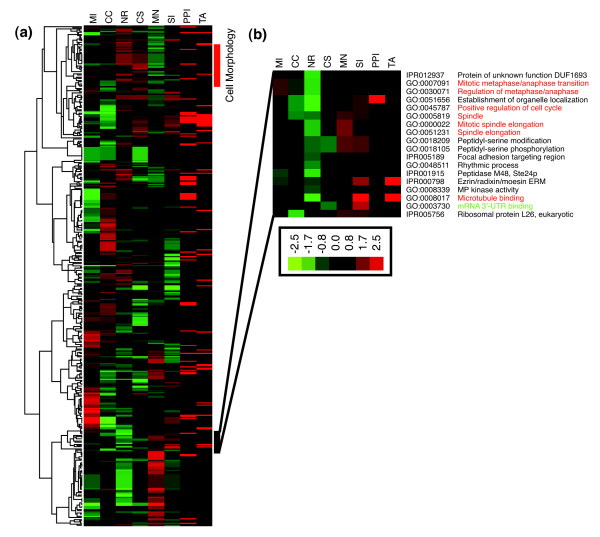
OPI clustering of high-content screening (HCS) results based on GO/IPR annotations. **(a,b) **Ontology-based pattern identification (OPI) heat map for each of the morphologic properties recorded in the microscopy based screen. Each row represents a Gene Ontology (GO)/InterPro group consisting of a significant number of annotated genes that shared the representative morphologic profile (P ≤ 0.05). Red and green color represents high and low scores in the corresponding morphological parameters. Headings symbolize morphologic parameters for mitotic index (MI), cell count (CC), nuclear roundness (NR), cell shape (CS), multinucleation (MN), and spindle intensity (SI). Red bars in the PPI and TA columns represent statistically significant support for the gene group based on protein-protein interaction (PPI) and tissue expression (TA) databases; these are determined based on OPI meta-analyses exhibiting multiple protein interactions or mRNA co-expression across 79 tissue samples, respectively. Groups shown in red contain known mitotic or spindle regulation genes, whereas those shown in green contain interesting novel components.

As expected, genes previously annotated as having mitotic, cell cycle, and cell division roles clustered together tightly. The GO molecular function enrichment clearly identified the following GO terms: sister chromatid segregation (*P *= 10^-5.2^), mitotic metaphase/anaphase transition (*P *= 10^-6.8^), and mitotic spindle elongation (*P *= 10^-6.1^), among others (Figure 2b). However, several other GO/InterPro groups also clustered with these known mitotic players, including genes for RNA binding, splice regulation, and RNA localization. This observation has recently been supported by the discovery that RNA-dependent complexes have a direct role in regulating microtubule dynamics independent of translational activities [20]. In fact, two of our strongest mitotic blocks occurred as a result of silencing *SMU-1*. The *C. elegans *genomic homolog of *SMU-1 *encodes a putative RNA-binding protein that has been implicated in pre-mRNA splicing [21]. SMU-1 has been shown to co-purify with the 45S spliceosome complex [22]. Recently, a similar study looking at cell cycle factors [23] also identified RNA-splicing machinery components as playing a role in mitotic spindle assembly.

The OPI pattern analysis provided an unbiased and systematic functional classification of the screening dataset. More importantly, a careful study of the resulting clusters enabled us to prioritize an additional 50 interesting siRNAs that were missed by the arbitrary 3σ cut-off. Two genes in particular were identified using this approach, namely *IKBKB/IKK2 *and *NFKBIA/IκBα*. These members of the nuclear factor-κB pathway are predicted to play a role in spindle functions using our OPI analysis and are described elsewhere [24,25].

**Figure 3:**
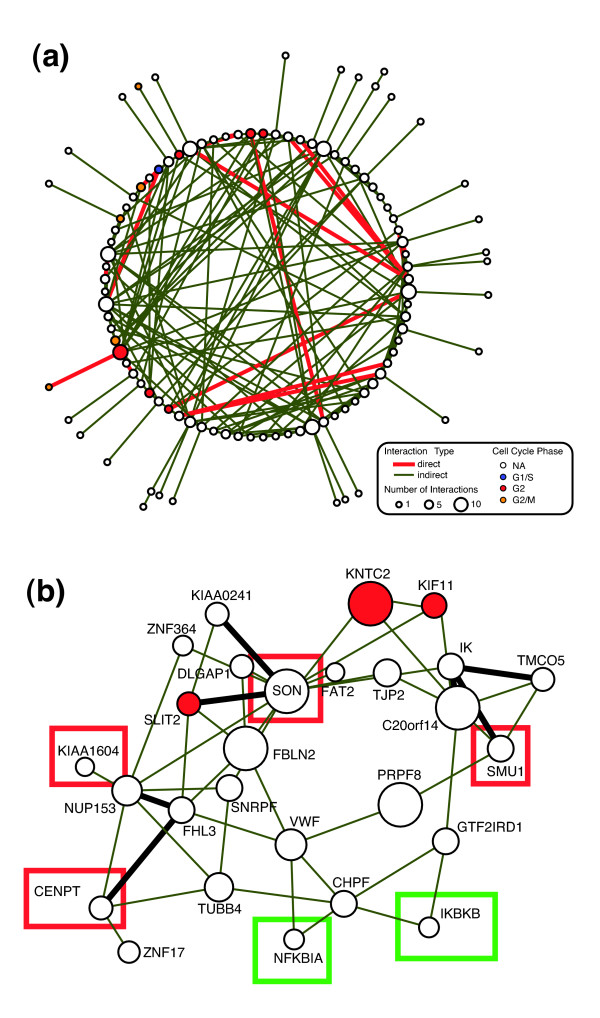
Validation of siRNA sequences for essential mitotic genes. **(a) **Protein-protein interaction (PPI) network for candidate genes in which at least one direct interaction (red lines) or indirect interaction (green lines) was identified. The cell cycle phases, based on previous expression analysis, are mapped on each protein bubble based on color. **(b) **PPI network for SON DNA/RNA-binding protein identified a number of novel components. Proteins in red squares, validated in our follow-up studies, included KIAA1604, FLJ13111/CENP-T, SON, and SMU-1. The green squares represent the nuclear factor-κB proteins examined elsewhere [25].

In addition to the GO analysis that profiled our 276 candidate genes as involved in mitosis, chromosome segregation, and spindle-related pathways, we further assessed whether the function of novel genes could be elucidated by combining data from previous cell cycle studies with the PPI data. (See Additional data file 4 for the complete list of all genes grouped in each mitotic/spindle related cluster.) Whitfield and coworkers [26] previously identified a list of 1,134 human cell cycle genes and assigned them to each cell cycle phase based on a global gene expression profile study. Among them, 748 genes were targeted in our siRNA collection. Statistical analysis showed that our list of mitotic genes is highly enriched in these cell cycle related elements (*P *= 10^-3.3^), especially those genes implicated in prometaphase (*P *= 10^-4.8^) or metaphase (*P *= 10^-3.1^). The analysis of the PPI database for the 276 candidate genes resulted in 110 proteins forming 213 interactions (17 direct and 196 indirect) with a *P *value of 0.0002 based on iterative simulations (Figure 3a). (See Additional data file 5 for an enlarged version of the connectivity map.) Given 276 randomly selected siRNAs, only one out of 5,000 simulation runs can produce a network consisting of at least 110 nodes. This implies our PPI network is unusually rare (*P *= 10^-3.7^) and further supports the novel protein interactions identified. One especially interesting subset of the interaction network suggested that novel proteins, such as KIAA1604, may interact with known spindle regulators such as Kif11/Eg5 (Figure 3b). Kif11 is a bipolar microtubule (MT) motor protein with an important role in spindle function [27].

**Figure 4:**
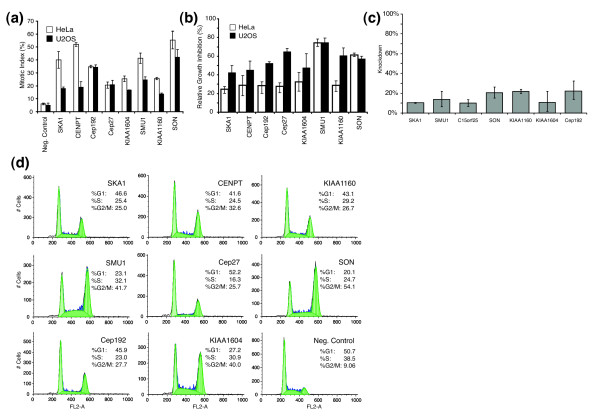
Cell cycle validation of isolated genes. **(a) **Quantitative comparison of mitotic index values based on phospho-histone H3 (pHis) detection in HeLa and U2OS cell lines. **(b) **Proliferation analysis. Cell counts after 48 hours of small interfering RNA (siRNA) incubation are determined and proliferation is reported as the ratio of cell counts compared with those from the negative control. **(c) **mRNA knockdown levels were determined using quantitative polymerase chain reaction and are reported as a percentage of target mRNA when cells are transfected with a negative control siRNA. (d) Cell cycle analysis of HeLa cells treated with siRNAs to determine relative G1, S, and G2/M cells per candidate gene validation using flowcytometry.

Because siRNAs can inhibit the expression of nontargeted genes, we first went about confirming the mitotic function of these genes by designing additional unique and nonoverlapping sequences directed against the original genomic targets. Because of cost limitations, 15 genes were selected from our candidate list and additional siRNA sequences designed. Among these, eight genes confirmed the previous observation in two different cell lines (HeLa and U2OS). Thus, we ultimately confirmed at least three different siRNA sequences per gene yielding the same phenotype. The strongest of these were then used for further analysis (Figure 4a and Table 1). Two of these genes encoded either hypothetical or completely novel open reading frames. We also observed that these siRNAs caused a significant reduction in cellular proliferation when compared with the negative control, further supporting the mitotic arrest phenotype (Figure 4b). The effective silencing capacity of the siRNAs was also examined using reverse transcription polymerase chain reaction to ensure knockdown of our directed target. The most effective siRNAs reduced mRNA levels by 70% or greater for our validation studies and further suggested that the appropriate gene was being targeted (Figure 4c). These siRNAs were further analyzed using flow cytometry on transfected cells to establish a more complete cell cycle examination. The G_2_/M peaks, representing a 4n cellular DNA content, were more pronounced than the negative control in all eight cases (Figure 4d). Taken together, these findings strongly support that our candidate list is highly enriched in components that play an essential role in mitotic cell cycle progression.

**Table 1:** Validated siRNA sequences

Number	Gene name	Gene ID	siRNA Sense Sequence1	siRNA Sense Sequence2	siRNA Sense Sequence3
1	SKA1/C18orf24	NM_001039535	CCUUCGUACAUGAAAUCCCGCUUAA	CGCUUAACCUAUAAUCAAA	GGAGUUCACAACUUUGAAA
2	CENPT/C16orf56	NM_025082	GACGAUAGCCAGAGGGCGUUU	GGAGGUAUUUGCUGCUCAU	AGUAGUGGCCAGGCUUCAA
3	KIAA1160	NM_020701	GGCUCAGAUUCAGAAUGCUGGUUUA	AGCAGAAGUUCAUUGCUCA	
4	SMU1	NM_018225	GAGGAGACUACUGUGUCUCUGAAUA	GCUCGAAAGCAUGUACUUA	GUGAAGAUGGACUCCUAAA
5	Cep27/C15orf25	NM_018097	UGAACUUCACCAGACUACAGCAGAU	GCUUUAUAUUGGAGUAUCA	GCUGUUUAUCACAGAUAUA
6	SON/C21orf50	NM_138925	GAUGUCAUCUUAUACUGCUGAUCGU	GGUCUUUCGUGGUCAGUAA	
7	Cep192	NM_032142	AGCAGCUAUUGUUUAUGUUGAAAAU	CAGAGGAAUCAAUAAUAAA	GGUGGAGAUGAAAUUUCAA
8	KIAA1604	NM_020943	GCAUGAAACUCUGCAGCCAUUCUUU	GAACGAAAUAACUACAGUA	

Table contains all of the validated sequences identified during either the screen or the validation process. siRNA, small interfering RNA.

**Figure 5:**
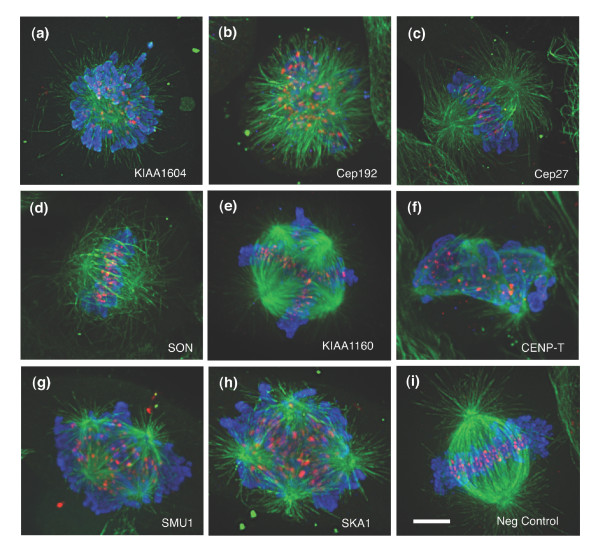
Spindle defects observed for RNAi phenotypes using confocal fluorescence microscopy. Cells transfected with single (double-stranded) small interfering RNA targeting **(a) ***KIAA1604*, **(b) ***KIAA1569/Cep192*, **(c) ***FLJ10460/Cep27*, **(d) ***SON*, **(e) ***KIAA1160*, **(f) ***FLJ13111/CENP-T*, **(g) ***SMU-1*, **(h) ***C18orf24/SKA1*, and **(i) **negative control. Cells were stained for α-tubulin (green), phospho-histone H3 (pHis; blue), and the CREST protein (red). Scale bar represents 5 μm.

To elucidate further the nature of the mitotic cell cycle arrests observed, we examined whether these genes directly impinged on spindle organization. Using confocal fluorescence microscopy, we closely examined both the spindle and chromatin organizations in the siRNA treated cells and discerned a spectrum of spindle defects (Figure 5a-h). The RNAi phenotypes demonstrated distinct spindle alterations: aberrant MT organization, different spindle size, and abnormal centrosomal numbers.

Interestingly, several of the proteins predicted as playing a role in sister chromatid segregation based on our OPI/InterPro analysis exhibited strong centrosomal functions. Centrosomes duplicate before the onset of mitosis and provide an essential MT organization function during spindle assembly [28]. Silencing of either *KIAA1569/Cep192 *or *KIAA1604 *generated monopolar spindles with a high number of cells exhibiting MTs emanating from a single foci (Figure 5a,b). *KIAA1604 *encodes a hypothetical protein with predicted protein and RNA binding domains. We have also recently shown that KIAA1569/Cep192 localizes to the centrosomes and plays an important role in the assembly and function of mitotic centrosomes [29]. Conversely, downregulation of another previously identified centrosomal protein, FLJ10460/Cep27, yielded a very different spindle organization [30]. Cep27 depleted cells formed a relatively normal looking bipolar spindle, with the chromosomes aligned along a metaphase plate (Figure 5d). When these images were compared with wild-type mitotic spindles (Figure 5i) we observed that the MT organization around the ends of the spindle did not form discrete foci. The high number of pHis positive cells (about 18% ± 4%; Figure 4a) implies that they are incapable of surpassing the metaphase to anaphase transition.

The SON DNA/RNA binding protein was also confirmed as having an effect on spindle structure in our studies. We observed that the spindles in *SON *siRNA treated cells were highly shortened (Figure 5d). This particular phenotype appeared similar to Kid/kinesin-10 depleted spindles [31]. Kid is a chromokinesin motor protein that has both MT-binding and DNA-binding domains [32]. These data suggests that SON works in combination with Kid.

Inhibiting the expression of the hypothetical gene *KIAA1160 *yielded a clear tetrapolar organization. We observed that the chromatin commonly organized into four to six metaphase plates per cell (Figure 5e). However, this multipolar arrangement differed from *FLJ13111/CENP-T*, *SMU-1*, and *C18orf24/SKA1 *knockdowns, all of which showed highly disorganized spindles (Figure 5f-h). CENP-T was recently identified as a component of the CENP-A nucleosome-associated complex, whereas SKA1 has been identified as a spindle and kinetochore associated protein [33,34]. The depletion of these proteins resulted in sparse MT arrays that often exhibited twisted or bent spindles. The chromosomes appear to interact with the spindle MTs but failed to organize into metaphase plates. This particular aberrant phenotype often resembled Rae1-depleted spindles. Rae1 was previously characterized as a RNA-MT binding protein whose role in spindle assembly was originally elucidated in *Xenopus *extracts [20]. In addition, *C18orf24/SKA1 *and *FLJ13111/CENP-T*, were also assigned into ribonucleoprotein groups (IPR001163 and IPR006649, respectively) and clustered near the sister chromatid segregation in the OPI/InterPro analysis (Figure 2a).

**Figure 6:**
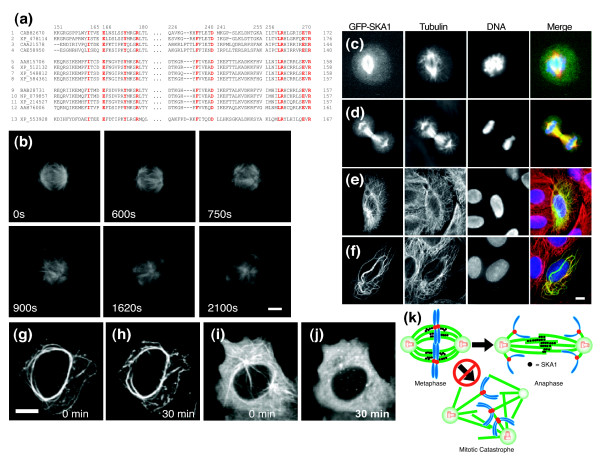
Analyzing the role of SKA1 in MT dynamics. **(a) **DUF1395 domain sequence comparison showing highly conserved amino acids (red) across multiple organisms: CAB82670 (Arabidopsis thaliana), XP_478114 (Oryza sativa), CAA21578 (Caenorhabditis elegans), CAE58950 (Caenorhabditis briggsae), AAH15705 (Homo sapiens), XP_512132 (Pan troglodytes), XP_548812 (Canis familiaris), XP_584361 (Bos Taurus), BAB28731 (Mus musculus), NP_079857 (Mus musculus), XP_214527 (Rattus norvegicus), AAH76006 (Danio rerio), and XP_553928 (Anopheles gambiae str). **(b) **Time-lapse microscopy monitoring spindle assembly in U2OS cells for 2,100 seconds. Scale bar: 3 μm. Panels c to f show green fluorescent protein (GFP)-SKA1 localization in **(c) **metaphase, **(d) **anaphase, and **(e,f) **interphase. **(f) **Localization of GFP-SKA1 in interphase cells over-expressing GFP-SKA1 for more than 24 hours. Scale bar: 5 μm. Panels g and h show GFP-SKA1 localization in transfected HeLa cell **(g) **before and **(h) **30 minutes after 10 μmol/l nocodozole treatment; **(i,j) **negative control images. Scale bar: 5 μm. **(k) **Model for SKA1's role in maintaining spindle integrity. SKA1 bundles microtubules (MT) and generates thicker and stronger fibers. Therefore, it prevents the loss of the spindle integrity before onsent of anaphase. Loss of this activity results in aberrant spindles with more than two poles.

We searched for evidence of potential functions for homologs of our novel genes in other organisms. Although none had been implicated as playing a role in spindle assembly, *SKA1 *is highly conserved among metazoans. The *SKA1 *gene encodes a 255-amino-acid protein with an evolutionarily conserved yet uncharacterized domain, DUF1395, that hinted at its fundamental importance [35] (Figure 6a).

We have also observed that SKA1 is required for maintaining spindle integrity during bipolar assembly. The presence of four or more microtubule organizing centers (MTOCs) per cell led us to wonder at what point the spindle assembly failed in the absence of SKA1. We performed time lapse microscopy on HeLa and U2OS cells stably expressing green fluorescent protein (GFP)-α-tubulin and transfected with the siRNA of *SKA1*. When we monitored the progression of the fluorescent spindles during assembly (Figure 6b) we noticed that, in many cases, a bipolar spindle begins to form but rapidly degrades before the onset of anaphase. (See Additional data file 6 to watch the live cell movie of the *SKA1 *knockdown effects on spindle progression.) The ends of the spindle appear to drift away from the central portion of the spindle as though the integrity of the spindle was lost. After this initial disruption, the spindles underwent a number of rearrangements and ultimately reorganized as multipolar arrays, suggesting that SKA1 plays an important role in the transition of a metaphase to anaphase spindle arrangement.

To assess further the function of SKA1, we over-expressed an amino-terminal GFP tagged version, *GFP-SKA1*, and examined the localization of this protein in mitotic and interphase cells. During mitosis, GFP-SKA1 localizes in the central spindle fibers but not to the astral MTs. In interphase, it exhibits a pattern very similar to α-tubulin (Figure 6c-f). Surprisingly, when over-expressed for more than 24 hours in interphase cells, both the GFP-SKA1 and α-tubulin exhibited much longer and thicker MT bundles (Figure 6g,h). (See Additional data file 7 to watch the live cell movie of the *SKA1 *over expression results.) Our data suggest that SKA1 plays an important role in strabilizing MTs. To investigate how upregulation of *SKA1 *was affecting MT stability, we examined the effects of treating SKA1 over-expressing cells with 10 μmol/l nocodazole for 30 minutes (Figure 6e,f). Even in the presence of the strong MT destabilizing agent, we still observed the presence of thick MT bundles.

The protein localization, live cell analysis, and nocodazole resistance studies support a role for SKA1 in MT bundling and stability. The disorganized spindle arrangements demonstrate that SKA1 clearly plays an important role in maintaining the spindle's structural integrity. We also noticed that GFP-SKA1 localizes to the central spindle fibers, further suggesting a role for this protein in MT stabilization. We speculate that the bundling properties of the protein may strengthen the spindle by resisting the tensile forces between the spindle poles. The additional strength of multiple MTs working in concert may create stronger fibers that are essential for maintaining spindle integrity (Figure 6k).

## Conclusion

In summary, this study describes a methodology for the identification of functionally relevant activities in large-scale RNAi datasets, and provides molecular insights into the fundamental process of mitosis and chromosome segregation. Our global profiling of statistically and biologically correlated morphologic patterns enabled us to predict functional roles of novel genes and has provided us with a more complete inventory of spindle components. To predict the specific function of these novel genes, we performed exhaustive bioinformatic analysis of these data. Using OPI algorithms, we clustered these genes in different functional families. Moreover, the aberrant multipolar spindles exhibited similar phenotypes with Rae1 depleted spindles. These data suggest that SMU-1 may also play an important role in spindle assembly. The preponderance of spliceosome or pre-mRNA components further raises the question regarding the exact role that mRNAs play in spindle functions and whether specific mRNA processing activities are required for spindle ribonucleoprotein complexes. Future studies will focus on elucidating what role these proteins play in the formation of bipolar spindles.

## Materials and methods

### Genome-wide siRNA library screen

The siRNA library of 49,164 double-stranded oligonucleotides (21-mers) were synthesized with two different siRNAs per gene (Qiagen, Germantown, MD, USA). This library would allow us to inhibit the expression of 23,835 genes. These two siRNAs per gene were pooled and arrayed into 384-well plates with 7 ng of each siRNA/well. The library was (retro)transfected by incubating the pre-arrayed siRNAs in 20 μl serum-free OptiMem cell culture media (Invitrogen, Carlsbad, CA, USA) containing 40 nl Lipofectamine2000 (Invitrogen). Twenty microliters of Dulbecco's modified Eagle's medium (Invitrogen) supplemented with 10% fetal bovine serum (FBS; Hyclone, Logan UT, USA), penicillin-streptomycin-glutamine (Invitrogen), and 1.5 × 10^6 ^HeLa cells/ml (about 2,000 cells/well). The plates were placed in a humidified chamber (5% carbon dioxide, 37°C) for 48 hours. After incubation, the majority of the media was aspirated from the wells and the cells were fixed using -20°C methanol and treated based on methods described previously [36]. For the analysis of mitotic indices, the fixed cells were fluorescently labeled using the anti-phospho-Histone H3 (Ser10) Mitosis Marker (Upstate, Waltham, MA, USA), and as a secondary antibody we used the anti-rabbit (Alexa647) antibody (Molecular Probes, Eugene, OR, USA). Cells were also stained with the mouse monoclonal anti-α-tubulin-FITC DM1A antibody (Sigma-Aldrich, Saint Louis, MO) and Hoechst 33342 for detecting DNA. In these plates we also performed cell count and quantified spindle intensity. For cellular morphology and multinucleation status, cells were labeled with the CellTrace Far Red DDAO-SE and Hoechst 33342 dyes (Molecular Probes). Both types of analysis were run in tandem.

### Multi-parametric image based screen to identify genes involved in mitotic progression

The effect of each siRNA gene set on mitotic progression was accurately determined by analyzing approximately 1,000 cells per target gene and carried out in duplicate to limit experimental errors. Quantitative image analysis was designed to measure levels of pHis on a single cell basis and achieved using a class of image-segmentation techniques for morphologic and cellular identification. Each plate included an Alexa647-labeled negative control siRNA to establish a baseline mitotic index (around 5%) and to determine transfection efficiencies, which are typically 95% (data not shown). In addition to the mitotic index, we also similarly analyzed the number of cells, the spindle intensity, and morphologic parameters: nuclear roundness, cell shape, and multinucleation.

### Quantitative phenotypic analysis

For the quantitative image analysis, multiple exposures per well were acquired using the Opera automated high-content screening microscope (PerkinElmer, Hamburg, Germany) and analyzed using the Acapella software program (PerkinElmer). The fluorescent nuclear channel (Hoechst 33342 signal) is converted into a binary image by thresholding the gray-scale image and used as a mask for discriminating between nuclei. Once binarization is complete, regions of interest (ROIs) are established using contour-based detection to identify the edge of nuclei. Each edge map is converted into a geometric feature and designated as a nuclear ROI. These regions are filtered based on size and fluorescent intensity to remove ROIs from non-nuclear signals sometimes created from particulate or fluorescent cellular debris. The mitotic index per well is established by measuring the average fluorescent intensity in the overlapping phospho-histone channel (Alexa647 signal) and gating on only those nuclear ROIs that have a positive pHis signal. Similar procedures were used to quantify the number of cells and the different morphologic parameters. To avoid plate to plate inconsistency, raw measurements were Log_2 _transformed and normalized based on the plate median. The data between replicate sets were averaged on a well by well basis. Positive wells were selected for having a value greater than three standard deviations from the screen mean.

### OPI analysis of morphologic profiles and candidate selection

The algorithm and application of OPI clustering are described in detail in previous reports [18,37]. The algorithm is initiated using three genes annotated to be members of a protein family (for instance, *PLA1A*, *PNLIPRP1*, and *PNLIPRP2*; *IPR000734 *in InterPro). By examining the morphologic profiles of these three genes, an 'average' profile best representing the proteins is automatically constructed, against which all genes in the dataset are ranked according to the similarities measured by either Pearson correlation coefficient or a Euclidean metric. We assume that genes ranked near the top are more likely to be associated with this family. The OPI algorithm iteratively descends the rank list and tracks the number of genes already annotated and those not yet annotated, from which the false discovery rate and true positive rate can be estimated by conservatively assuming all un-annotated genes are false positives. The algorithm also calculates an accumulative hypergeometric *P *value that represents the odds of un-annotated genes in the resultant cluster sharing a similar profile to the annotated genes by chance. A lower *P *value indicates a more significant functional enrichment. OPI iterations stop when the optimal (minimum) *p *value is found.

From the example given above, IPR000734, we obtained a cluster of ten genes, among which three are known lipase proteins, seven have limited InterPro annotations, and three have no annotation. These gave a *P *value of 10^-9.5^, a true positive rate of 100%, and a false discovery rate of 57% (4/7). Because we do not expect that morphologic profiles of only six parameters will enable accurate gene function prediction, the purpose of the OPI analysis here is mainly to obtain the *P *value in order to validate the assumption that genes sharing similar morphologic profiles tend to share similar functions.

In the work described here we applied the OPI analysis to 4,660 Gene Ontology (GO) terms and 3,196 InterPro terms that contain at least two siRNAs in the current screening collection, and obtained one resultant cluster per knowledge term. To establish the correlation among genes, GO, and IPR groups, the procedure was then repeated 100 times on randomized datasets. If the permutation simulations resulted in the same or better *P *value more than 5% of the time, then the original cluster was rejected. Finally, 445 clusters survived the permutation test (permutation *P *value ≤ 0.05); their morphologic profiles were then hierarchically clustered to give a systematic overview of the biologic processes involved in cell cycle (Figure 2a). All 445 clusters and their key output parameters are available in Additional data file 3.

Each OPI cluster contains both annotated genes as well as novel candidates predicted based on the commonly shared morphologic profile. The gene list was then used as a hypothetical ontology term and we repeated the OPI procedure on both the Prolexys human PPI database [38] and GNF human tissue database (79 tissue samples used) [19] and unveiled additional statistical evidence to support the quality of the OPI clusters. Because our original 226 siRNA hits rely on an arbitrary cut-off value on mitotic index score (≥ +3σ), OPI clusters include potential false negative hits that have interesting morphologic profiles but mitotic index score below the cut-off. We therefore hand selected an additional 50 siRNAs from the OPI clusters, and that resulted in a total of 276 siRNA candidates.

### Statistical analysis of screening hits

The GO/InterPro function enrichment analysis was carried out on the above mentioned candidate list using the standard statistical tests, in which *P *values were estimated using hypergeometric distribution. The best *P *value (10^-7.3^) is obtained for term GO:0007067 - mitosis. There are 55 functional groups scored with a *P *value > 0.01. (See Additional data file 4 for the complete data table.) Our siRNA collection contains 748 genes that have cell cycle phase assigned by Whitfield and coworkers [26] based on their mRNA gene expression profiling study. Similar analysis using hypergeometric distribution showed our hit lists contain 22 cell cycle genes (*P *= 10^-3.3^), with 11 in G_2 _phase (*P *= 10^-4.8^) and nine in G_2_/M phase (*P *= 10^-3.1^). We retrieved all the direct and indirect (via one nonhit protein) protein interactions among the hit members from a two hybrid database (Prolexys), with the requirements that each edge must have a minimum confidence score of 1.0.

### Imaging of living cells

HeLa and U2OS cells where stably transfected with a plasmid encoding the sequence or α-tubulin amino-terminal fused to GFP (BD Bioscience, San Jose, CA Clontech Cat. 632349). These cells were reverse transfected with the siRNA of SKA1 or a control siRNA. In brief, 80 nmol of the siRNA was diluted in 500 μl of OptiMem (Invitrogen) and placed in a 35 mm glass bottom dish (MatTek Corporation, Ashland, MA). Two microliters of Lipofectamine2000 (Invitrogen) diluted in 500 μl of OptiMem were added to the dish and the complexes were incubated for 1 hour. We plated 2.0 × 10^5 ^cells diluted in 1 ml Dulbecco's modified Eagle's medium supplemented with 10% FBS, and 12 hours later we added additional FBS to a final concentration of 10%. Forty-eight hours after the transfection, the cells were placed in L-15 media (Gibco, Carlsbad, CA) and imaged using an Ultraview RS spinning disk confocal microscope (PerkinElmer), with a controlled-temperature stage, allowing prolonged fluorescent analysis of human live cells. Z-series of images through the entire cell were acquired and displayed for analysis as multiple intensity projections. For live cell movies, one z-series of images was acquired every 60 seconds.

## Abbreviations

FBS, fetal bovine serum; GFP, green fluorescent protein; GO, Gene Ontology; OPI, ontology-based pattern identification; MT, microtubule; pHis, phosphorylated form of histone H3; PPI, protein-protein interaction; RNAi, RNA interference; ROI, region of interest; siRNA, small interfering RNA.

## Authors' contributions

DRR, PD, and SG performed the siRNA library transfections and high-content imaging of the entire collection. DRR wrote the image analysis and initial mitotic index algorithms using the Acapella language (Perkin Elmer). DRR also completed all of the flow cytometry, quantitative polymerase chain reaction, and cellular proliferation experiments and analyses. MAG-F and DJS conducted the high-resolution and live cell microscopy experiments. YZ and SB provided the statistical analysis using the OPI clustering and InterPro interaction network methods. ML, DH, CM, JH, MR, FN, and JL are responsible for the siRNA sequence design and production of the genome-wide library. SKC and JSC provided technical expertise and intellectual direction. All authors, along with the GNF and Novartis legal departments, have approved the manuscript.

## Additional data files

The following additional data files are available with the online version of this paper. Additional data file 1 is a list of candidate genes from our initial high-content and OPI analysis. Additional data file 2 is a figure with additional images and parameters used for the image-based morphologic analysis. Additional data file 3 is a data table showing all the OPI clusters. Additional data file 4 is a data table listing the individual genes associated with each mitotic cluster. Additional data file 5 is an enlarged version of PPI map for clarity. Additional data file 6 is a movie file showing the spindle defect when SKA1 is knocked down. Additional data file 7 is a movie file showing the effects of SKA1 over-expression.

## Supplementary Material

Additional data file 1 Presented is a data file showing the complete list of candidate genes isolated from our initial mitotic index thresholding and OPI clustering results. Click here for file

Additional data file 2 **(A) **Loss of spindle surveillance mechanisms or defects in cytokinesis may result in multinucleated cells. Thus, we also identified genes involved in checkpoint-independent spindle functions by analyzing changes in the cell populations based on their multinucleation status. We acquired an additional 308,736 images using a far-red cytoplasmic dye (DDAO-SE) to determine the number of discrete nuclei per cell (A, inset). For our analysis, images were segmented into ROIs based on each cell's cytoplasmic intensity. Image analysis of the ROIs then determined the number of discrete nuclei per cell. Using this approach we uncovered a number of siRNAs targeting known chromosomal passenger genes, such as INCENP, CDCA8, BIRC5, and AuroraB. Cytokinesis genes were also identified as an additional benefit of this approach, because failing to complete cell division can result in multiple nuclei per cell. Thus, we isolated MKLP-1, MgcRacGAP, and CIT along with other known cytokinesis members. **(B) **Shown is the nuclei DNA organization after the activation of apoptotic pathways resulting in noncircular shaped and fragmented nuclei patterns. **(C) **The approach that was used to fit nuclei and cell morphology pattern analysis. Those objects that have a poor fit are given a lower score. **(D) **Typical image of cytoplasmic and DNA channels of cells that are mostly rounded. **(E) **Graph illustrates the time dependent change in nuclear shape. Cells treated with PLK1 siRNAs undergo apoptotic events relatively soon after treatment and show a high degree of fragmentation. **(F) **Watershed analysis (cytoplasmic masking) and signal intensity measurement approach for spindle and microtubule intensity on a cell by cell basis. **(G) **Image segmentation used to identify individual nuclei for proliferation comparisons. Click here for file

Additional data file 3 The data table demonstrates all of the OPI clusters along, with calculated values listed. Click here for file

Additional data file 4 The data table lists the statistically significant clusters of genes associated with the mitotic/spindle processes. Click here for file

Additional data file 5 Enlarged version of the interaction map shown in Figure 3a, with protein labels added for reader's clarity. Click here for file

Additional data file 6 Provided is a sample live-cell movie of a mitotic cell transfected with an siRNA against SKA1 and expressing GFP-tubA1. The movie demonstrates the formation of a metaphase-like spindle before falling apart prior to the onset of anaphase. Click here for file

Additional data file 7 Provided is a sample live-cell movie of a HeLa cell transfected with the open reading frame for GFP-SKA1 driven by a cytomegalovirus promoter. Click here for file

## References

[B1] Scholey JM, Brust-Mascher I, Mogilner A (2003). Cell division.. Nature.

[B2] Compton DA (2000). Spindle assembly in animal cells.. Annu Rev Biochem.

[B3] Mitchison TJ, Salmon ED (2001). Mitosis: a history of division.. Nat Cell Biol.

[B4] Mishima M, Pavicic V, Gruneberg U, Nigg EA, Glotzer M (2004). Cell cycle regulation of central spindle assembly.. Nature.

[B5] Wittmann T, Hyman A, Desai A (2001). The spindle: a dynamic assembly of microtubules and motors.. Nat Cell Biol.

[B6] Carroll PE, Okuda M, Horn HF, Biddinger P, Stambrook PJ, Gleich LL, Li YQ, Tarapore P, Fukasawa K (1999). Centrosome hyperamplification in human cancer: chromosome instability induced by p53 mutation and/or Mdm2 overexpression.. Oncogene.

[B7] D'Assoro AB, Lingle WL, Salisbury JL (2002). Centrosome amplification and the development of cancer.. Oncogene.

[B8] Pihan GA, Purohit A, Wallace J, Malhotra R, Liotta L, Doxsey SJ (2001). Centrosome defects can account for cellular and genetic changes that characterize prostate cancer progression.. Cancer Res.

[B9] Sato N, Mizumoto K, Nakamura M, Maehara N, Minamishima YA, Nishio S, Nagai E, Tanaka M (2001). Correlation between centrosome abnormalities and chromosomal instability in human pancreatic cancer cells.. Cancer Genet Cytogenet.

[B10] D'Assoro AB, Barrett SL, Folk C, Negron VC, Boeneman K, Busby R, Whitehead C, Stivala F, Lingle WL, Salisbury JL (2002). Amplified centrosomes in breast cancer: a potential indicator of tumor aggressiveness.. Breast Cancer Res Treat.

[B11] Gadde S, Heald R (2004). Mechanisms and molecules of the mitotic spindle.. Curr Biol.

[B12] Wordeman L (2005). Microtubule-depolymerizing kinesins.. Curr Opin Cell Biol.

[B13] Bloom K (2005). Chromosome segregation: seeing is believing.. Curr Biol.

[B14] Bettencourt-Dias M, Giet R, Sinka R, Mazumdar A, Lock WG, Balloux F, Zafiropoulos PJ, Yamaguchi S, Winter S, Carthew RW, Cooper M, Jones D, Frenz L, Glover DM (2004). Genome-wide survey of protein kinases required for cell cycle progression.. Nature.

[B15] Chang P, Jacobson MK, Mitchison TJ (2004). Poly(ADP-ribose) is required for spindle assembly and structure.. Nature.

[B16] Morgan DO (1999). Regulation of the APC and the exit from mitosis.. Nat Cell Biol.

[B17] Chanda SK, White S, Orth AP, Reisdorph R, Miraglia L, Thomas RS, DeJesus P, Mason DE, Huang Q, Vega R, Yu DH, Nelson CG, Smith BM, Terry R, Linford AS, Yu Y, Chirn GW, Song C, Labow MA, Cohen D, King FJ, Peters EC, Schultz PG, Vogt PK, Hogenesch JB, Caldwell JS (2003). Genome-scale functional profiling of the mammalian AP-1 signaling pathway.. Proc Natl Acad Sci USA.

[B18] Zhou Y, Young JA, Santrosyan A, Chen K, Yan SF, Winzeler EA (2005). In silico gene function prediction using ontology-based pattern identification.. Bioinformatics.

[B19] GNF SymAtlas. http://symatlas.gnf.org/SymAtlas.

[B20] Blower MD, Nachury M, Heald R, Weis K (2005). A Rae1-containing ribonucleoprotein complex is required for mitotic spindle assembly.. Cell.

[B21] Spike CA, Shaw JE, Herman RK (2001). Analysis of smu-1, a gene that regulates the alternative splicing of unc-52 pre-mRNA in *Caenorhabditis elegans*.. Mol Cell Biol.

[B22] Makarov EM, Makarova OV, Urlaub H, Gentzel M, Will CL, Wilm M, Luhrmann R (2002). Small nuclear ribonucleoprotein remodeling during catalytic activation of the spliceosome.. Science.

[B23] Kittler R, Putz G, Pelletier L, Poser I, Heninger AK, Drechsel D, Fischer S, Konstantinova I, Habermann B, Grabner H, Yaspo ML, Himmelbauer H, Korn B, Neugebauer K, Pisabarro MT, Buchholz F (2004). An endoribonuclease-prepared siRNA screen in human cells identifies genes essential for cell division.. Nature.

[B24] Irelan J, Murphy T, Xu D, Gomez M, Zhou Y, DeJesus P, Rines DR, Verma IM, Sharp DJ, Tergaonkar V (2007). A role for IKK2 in bipolar spindle assembly.. Proc Natl Acad Sci.

[B25] Irelan JT, Murphy TJ, DeJesus PD, Teo H, Xu D, Gomez-Ferreria MA, Zhou Y, Miraglia LJ, Rines DR, Verma IM, Sharp DJ, Tergaonkar V, Chanda SK (2007). A role for IkappaB kinase 2 in bipolar spindle assembly.. Proc Natl Acad Sci USA.

[B26] Whitfield ML, Sherlock G, Saldanha AJ, Murray JI, Ball CA, Alexander KE, Matese JC, Perou CM, Hurt MM, Brown PO, Botstein D (2002). Identification of genes periodically expressed in the human cell cycle and their expression in tumors.. Mol Biol Cell.

[B27] Sawin KE, LeGuellec K, Philippe M, Mitchison TJ (1992). Mitotic spindle organization by a plus-end-directed microtubule motor.. Nature.

[B28] Kirschner M, Mitchison T (1986). Beyond self-assembly: from microtubules to morphogenesis.. Cell.

[B29] Gomez-Ferreria MA, Rath U, Buster DW, Chanda SK, Caldwell JS, Rines DR, Sharp DJ (2007). Human cep192 is required for mitotic centrosome and spindle assembly.. Curr Biol.

[B30] Andersen JS, Wilkinson CJ, Mayor T, Mortensen P, Nigg EA, Mann M (2003). Proteomic characterization of the human centrosome by protein correlation profiling.. Nature.

[B31] Tokai-Nishizumi N, Ohsugi M, Suzuki E, Yamamoto T (2005). The chromokinesin Kid is required for maintenance of proper metaphase spindle size.. Mol Biol Cell.

[B32] Shiroguchi K, Ohsugi M, Edamatsu M, Yamamoto T, Toyoshima YY (2003). The second microtubule-binding site of monomeric kid enhances the microtubule affinity.. J Biol Chem.

[B33] Foltz DR, Jansen LE, Black BE, Bailey AO, Yates JR, Cleveland DW (2006). The human CENP-A centromeric nucleosome-associated complex.. Nat Cell Biol.

[B34] Hanisch A, Sillje HH, Nigg EA (2006). Timely anaphase onset requires a novel spindle and kinetochore complex comprising Ska1 and Ska2.. Embo J.

[B35] Marchler-Bauer A, Bryant SH (2004). CD-Search: protein domain annotations on the fly.. Nucleic Acids Res.

[B36] Mitchison Lab Protocols. http://mitchison.med.harvard.edu/protocols.html.

[B37] Young JA, Fivelman QL, Blair PL, de la Vega P, Le Roch KG, Zhou Y, Carucci DJ, Baker DA, Winzeler EA (2005). The Plasmodium falciparum sexual development transcriptome: a microarray analysis using ontology-based pattern identification.. Mol Biochem Parasitol.

[B38] Prolexys Pharmaceuticals. http://www.prolexys.com.

