# Supplementary figures and images for: Whole genome functional analysis identifies novel components required for mitotic spindle integrity in human cells
Source: Genome Biol. 2008 Feb 26;9(2):R44. doi: 10.1186/gb-2008-9-2-r44 (PMC2374723; doi:10.1186/gb-2008-9-2-r44)

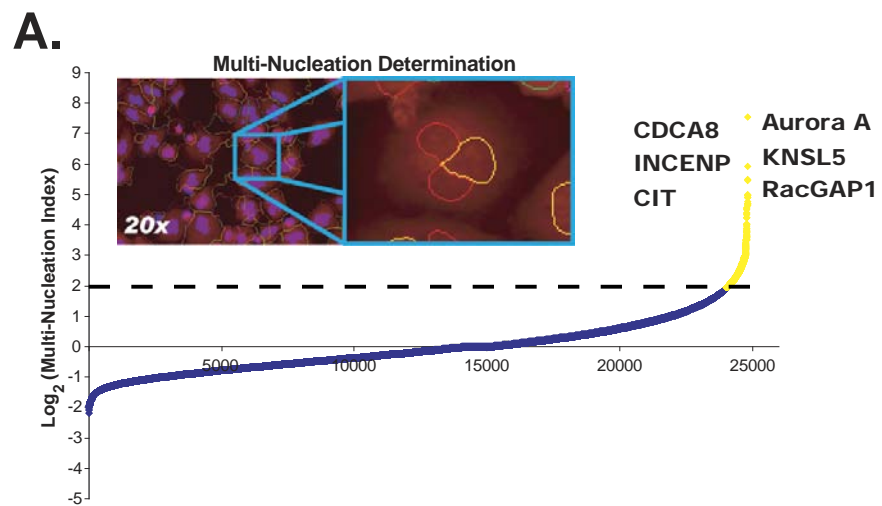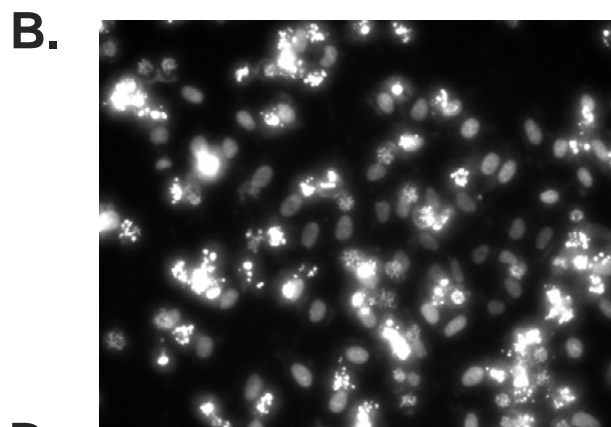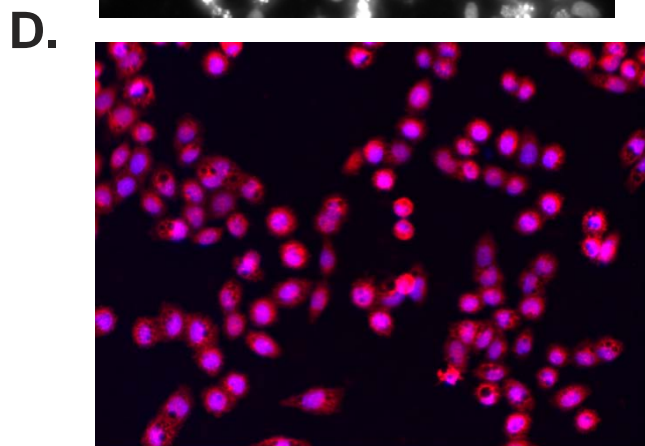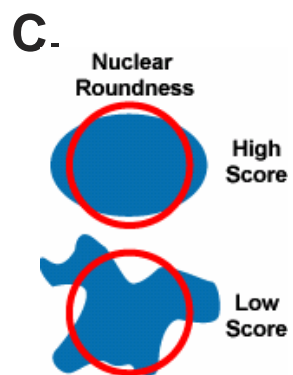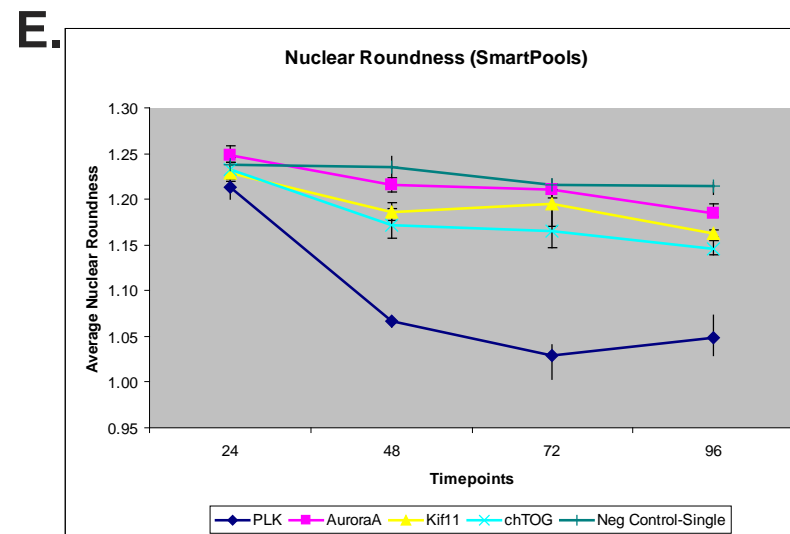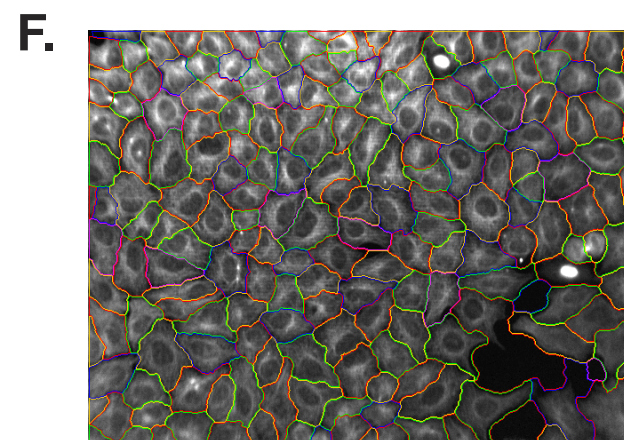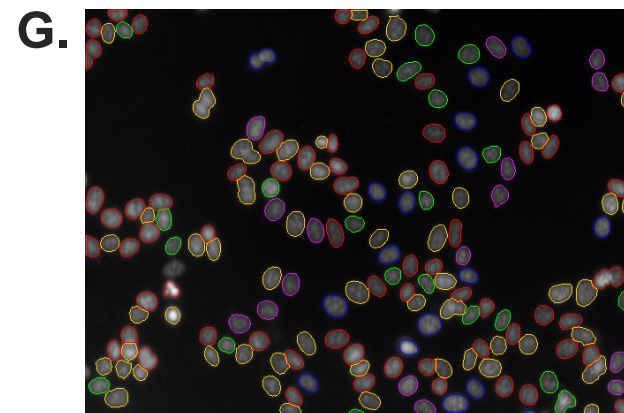

Figure S1

Supplement: Additional data file 2 — (A) Loss of spindle surveillance mechanisms or defects in cytokinesis may result in multinucleated cells. Thus, we also identified genes involved in checkpoint-independent spindle functions by analyzing changes in the cell populations based on their multinucleation status. We acquired an additional 308,736 images using a far-red cytoplasmic dye (DDAO-SE) to determine the number of discrete nuclei per cell (A, inset). For our analysis, images were segmented into ROIs based on each cell's cytoplasmic intensity. Image analysis of the ROIs then determined the number of discrete nuclei per cell. Using this approach we uncovered a number of siRNAs targeting known chromosomal passenger genes, such as INCENP, CDCA8, BIRC5, and AuroraB. Cytokinesis genes were also identified as an additional benefit of this approach, because failing to complete cell division can result in multiple nuclei per cell. Thus, we isolated MKLP-1, MgcRacGAP, and CIT along with other known cytokinesis members. (B) Shown is the nuclei DNA organization after the activation of apoptotic pathways resulting in noncircular shaped and fragmented nuclei patterns. (C) The approach that was used to fit nuclei and cell morphology pattern analysis. Those objects that have a poor fit are given a lower score. (D) Typical image of cytoplasmic and DNA channels of cells that are mostly rounded. (E) Graph illustrates the time dependent change in nuclear shape. Cells treated with PLK1 siRNAs undergo apoptotic events relatively soon after treatment and show a high degree of fragmentation. (F) Watershed analysis (cytoplasmic masking) and signal intensity measurement approach for spindle and microtubule intensity on a cell by cell basis. (G) Image segmentation used to identify individual nuclei for proliferation comparisons. [file gb-2008-9-2-r44-S2.pdf]

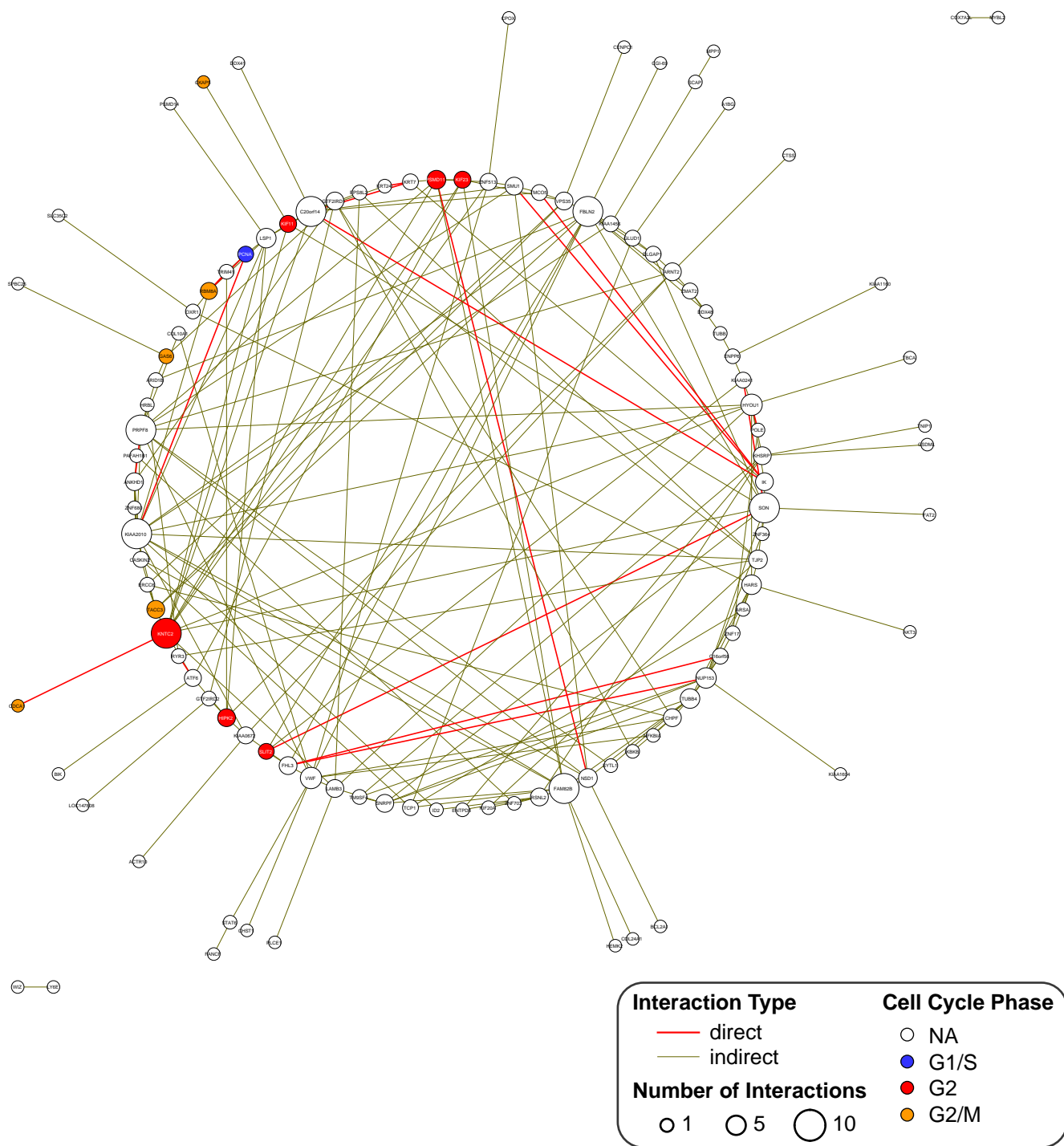

Figure S2

Supplement: Additional data file 5 — Enlarged version of the interaction map shown in Figure 3a, with protein labels added for reader's clarity. [file gb-2008-9-2-r44-S5.pdf]
